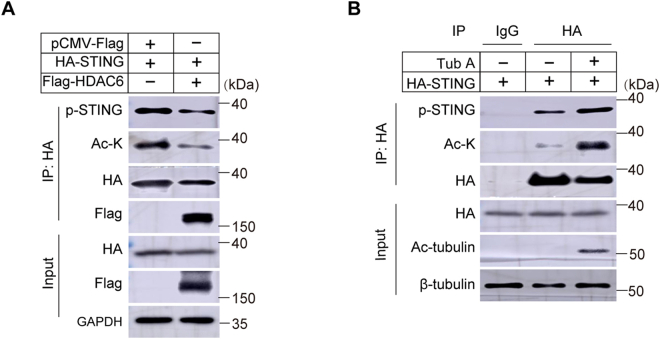# Histone deacetylase 6 inhibits STING-dependent antiviral immunity *via* site-specific deacetylation

**DOI:** 10.1016/j.jbc.2025.110841

**Published:** 2025-10-23

**Authors:** Min Qu, Dali Wei, Qunhua Ke, Pengyuan Cheng, Yiyuan Ma, Berihun Afera, Ke Guo, Miaomiao Li, Xiangping Yin, Xiangwei Wang, Jianlin Han, Yuefeng Sun

**Affiliations:** 1 State Key Laboratory for Animal Disease Control and Prevention, College of Veterinary Medicine, Lanzhou University, Lanzhou, China; 2 Gansu Province Research Center for Basic Disciplines of Pathogen Biology, Lanzhou, China; 3 State Key Laboratory for Animal Disease Control and Prevention, Lanzhou Veterinary Research Institute, Chinese Academy of Agricultural Sciences, Lanzhou, China; 4 College of Veterinary Medicine, Gansu Agricultural University, Lanzhou, China; 5 College of Animal Science and Technology, Nanjing Agricultural University, Nanjing, China; 6 Mekelle University, College of Veterinary Sciences, Mekelle, Tigray, Ethiopia; 7 Yazhouwan National Laboratory, Sanya, China

**Keywords:** HDAC6, STING, VSV, deacetylation, interferon, innate immunity

## Abstract

Histone deacetylase HDAC6 is a critical regulator of antiviral innate immunity, but its precise molecular mechanisms during RNA viral infection remain incompletely understood. In this study, we demonstrate that HDAC6 depletion (*via* siRNA knockdown or pharmacological inhibition) significantly suppresses vesicular stomatitis virus (VSV) replication. Further analysis revealed that HDAC6 modulates innate immune signaling by targeting the stimulator of interferon genes (STING) pathway, thereby attenuating type I interferon (IFN) responses. Mechanistically, HDAC6 directly interacts with STING and catalyzes its deacetylation at lysine 338 (K338). This post-translational modification impedes TBK1 recruitment by altering STING acetylation status, ultimately impairing STING phosphorylation at serine 366 (S366). Functional validation showed that overexpression of a STING with K338Q acetylation-mimetic mutant confers cellular resistance to VSV infection, establishing HDAC6-mediated STING deacetylation as a pivotal regulatory checkpoint in the antiviral response.

Microbial infections trigger innate immune and inflammatory responses, often leading to diseases. The initial defense is mediated by pattern recognition receptors (PRRs), including Toll-like receptors (TLRs), RIG-I-like receptors (RLRs), NOD-like receptors (NLRs), and C-type lectin receptors (CLRs), which detect pathogen-associated molecular patterns (PAMPs) (1, 2). In 2008, Ishikawa *et al*. identified stimulator of interferon genes (STING), a 379-amino-acid protein with five transmembrane motifs, as a key regulator of DNA virus-induced innate immunity (3). Subsequent studies established the cyclic guanosine monophosphate (GMP)-adenosine monophosphate (AMP) synthase (cGAS)-STING pathway as a central mediator of antiviral responses (4, 5, 6). Upon sensing cytosolic double-stranded DNA (dsDNA), cGAS synthesizes the second messenger 2′3′-cyclic GMP-AMP (2′3′-cGAMP), which binds and activates STING (5). Activated STING translocates from the endoplasmic reticulum (ER) to the Golgi, recruiting TBK1 and interferon regulatory factor 3 (IRF3) to induce type I interferons (IFNs) and inflammatory cytokines (7, 8, 9).

Emerging evidence suggests that STING plays a critical role in RNA virus infections. For instance, foot-and-mouth disease virus (FMDV) downregulates STING expression to evade immunity (10), and STING modulates host responses to SARS-CoV-2, HIV-1, HCV, ZIKV, and DENV (11). Post-translational modifications (PTMs) of STING, including ubiquitination and phosphorylation, are essential for its antiviral function. Recently, Beclin-1 was shown to acetylate STING at K338, triggering its autophagic degradation (12). However, the enzyme responsible for STING deacetylation remains unknown.

Histone deacetylases (HDACs) are classified into four classes: I (HDAC1, HDAC2, HDAC3, and HDAC8), II (IIa (HDAC4, HDAC5, HDAC7, and HDAC9) and IIb (HDAC6 and HDAC10)), III (sirtuins 1–7), and IV (HDAC11) (13, 14). Among them, HDAC6—a predominantly cytoplasmic enzyme with two deacetylase domains—has been extensively studied for its antiviral functions. HDAC6 deacetylates substrates such as TRIM21 (15), microtubules (MTs) (16), tubulin (17, 18, 19, 20), and β-catenin (21, 22, 23), modulating immune responses. However, some viruses, like influenza A virus (IAV), exploit HDAC6 to facilitate viral uncoating (24). Intriguingly, HDAC6 can translocate to the nucleus to regulate valine metabolism and DNA repair (25). Recent work revealed that HDAC6 deacetylates TRIM56, suppressing the cGAS-STING pathway during DNA virus infection (26). Nevertheless, its role in RNA virus replication and STING regulation remains unclear.

Tubastatin A (Tub A) is a selective HDAC6 inhibitor developed in 2010 (27). Tub A treatment inhibits viral replication, ameliorates liver ischemia/reperfusion injury (28, 29), attenuates HIV-1 Tat-induced inflammation (30), and suppresses hepatitis C virus replication (31).

VSV is an enveloped, single-stranded RNA virus that primarily infects livestock (cattle and horses), with sporadic zoonotic transmission to humans. Endemic to the Americas, VSV induces vesicular lesions in the oral cavity, hooves, and udders, posing substantial economic burdens to livestock farming. Its spike protein-mediated entry and host-dependent machinery have made VSV a model system for virology research. Notably, VSV’s rapid replication and broad tropism have enabled its development as a vaccine vector for pathogens like SARS-CoV-2 and Ebola virus (32, 33, 34). Additionally, its capacity for efficient gene delivery has spurred applications in gene therapy and oncolytic virotherapy (35, 36).

Here, we elucidate the role of HDAC6 in VSV infection. We show that HDAC6 inhibition suppresses VSV replication. Using mass spectrometry-based acetylome analysis, we identify STING as an HDAC6 substrate and demonstrate that HDAC6 deacetylates STING at K338, impairing TBK1 recruitment and STING phosphorylation at S366. Functional studies confirm that HDAC6-mediated STING deacetylation promotes VSV replication by dampening antiviral signaling.

## Results

### HDAC6 deacetylase activity is required for VSV replication

**Fig. S1:**
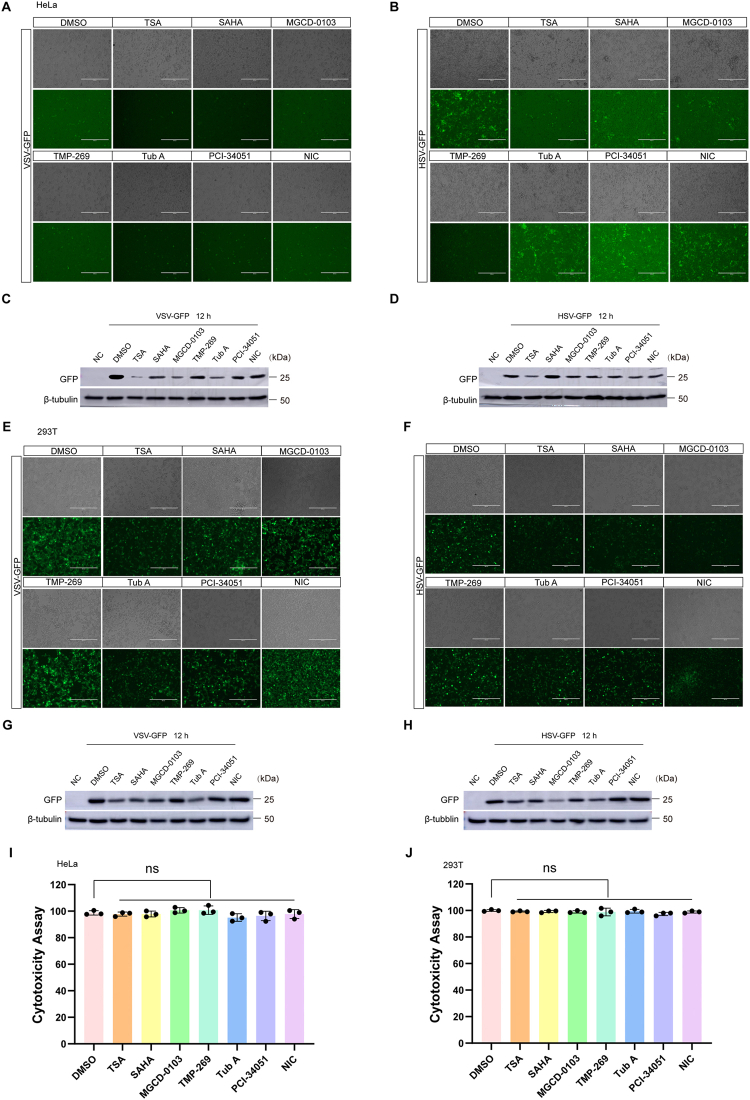


**Fig. S2:**
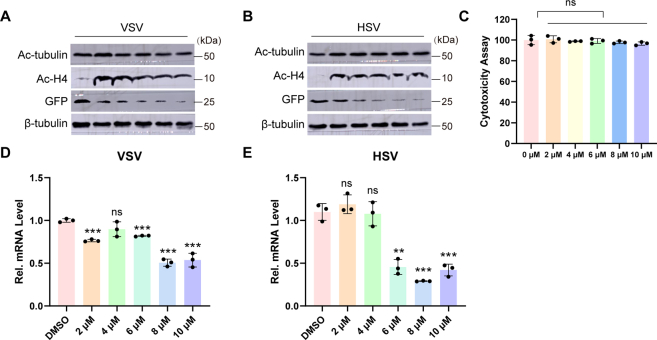


Previous studies have implicated HDACs in viral replication, infection mechanisms, and carcinogenesis (37). To assess the role of HDACs in viral replication, we treated cells with various HDAC inhibitors and monitored HSV-1-GFP (herpes simplex virus expressing green fluorescent protein, MOI = 10) and VSV-GFP (vesicular stomatitis virus expressing green fluorescent protein, MOI = 0.1) replication. Working concentrations of HDACs inhibitors (Table 1) were confirmed to be non-cytotoxic by CCK-8 assay (Fig. S1). Tub A significantly suppressed VSV replication (Fig. S1). In addition, we also found that MGCD-0103 inhibits the replication of HSV and VSV (Fig. S2).

**Table 1:** Working concentrations of HDACs inhibitors

HDACs inhibitors	Trichostatin A	Vorinostat	MGCD-0103	TMP269	Tubastatin A	PCI-34051	Nicotinamide
Targets	HDACs	HDACs	HDAC1	HDAC4	HDAC6	HDAC8	Sirtuins
			HDAC2	HDAC5			
			HDAC3	HDAC7			
			HDAC11	HDAC9			
Concentration	10 μM	10 μM	10 μM	10 μM	10 μM	5 μM	50 μM

**Figure 1:**
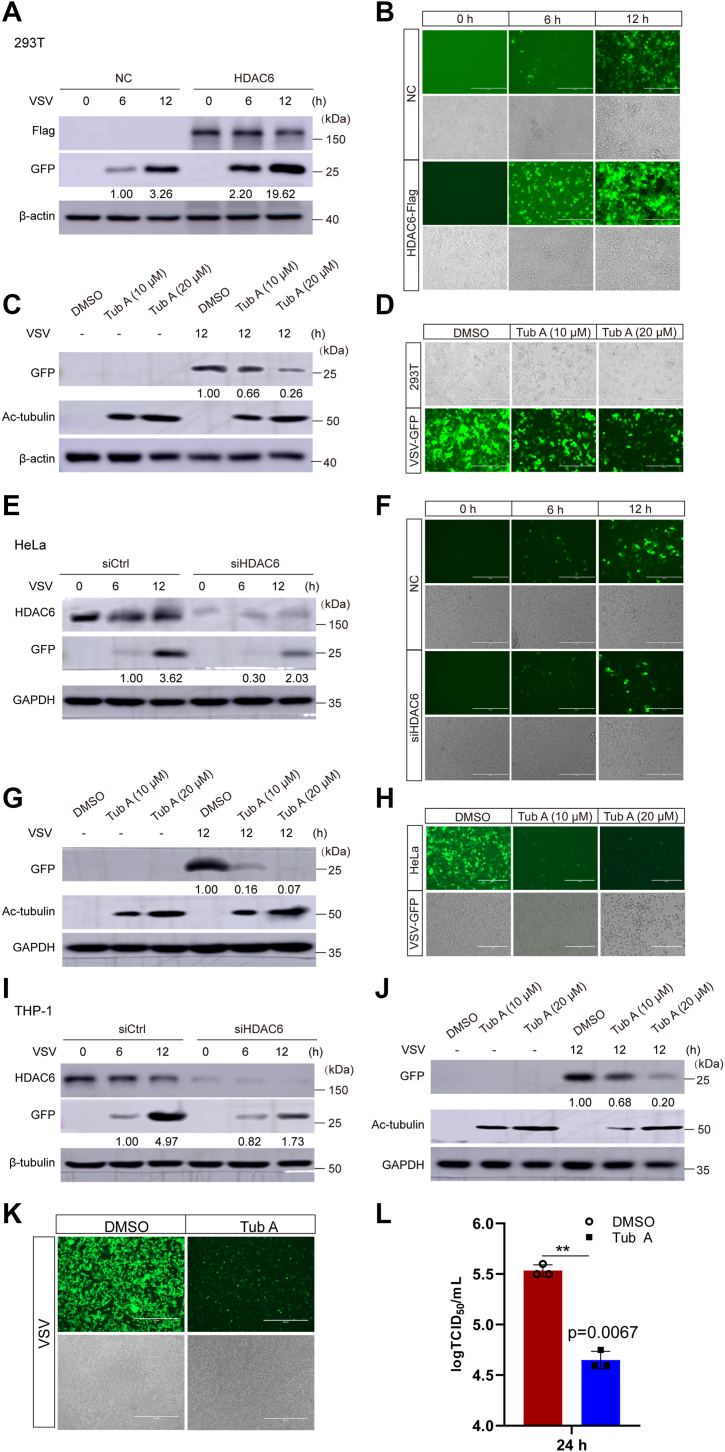
**HDAC6 knockdown or enzymatic inhibition suppresses VSV replication**. *A–B*, HEK293T cells were transfected with Flag-HDAC6 for 24 h, followed by VSV-GFP infection (0, 6, or 12 h). NC use pCMV-Flag empty vector as negative control. Viral replication was assessed by Western blotting and fluorescence intensity assays. *C–D*, HEK293T cells were pretreated with Tubastatin A (Tub A: 0, 10 or 20 μM, 24 h) before VSV-GFP infection. Ac-tubulin served as a positive control for Tub A activity. *E–F*, HeLa cells transfected with si-HDAC6 for 24 h were infected with VSV virus (0, 6 and 12 h), and viral replication was analyzed. SiCtrl use control siRNA as negative control. *G–H*, HeLa cells pretreated with Tub A (0, 10 or 20 μM, 24 h) were infected with VSV-GFP, and viral replication was measured. *I–J*, THP-1-derived macrophages with HDAC6 knockdown were treated with Tub A (0, 10 or 20 μM, 24 h) and infected with VSV for 12 h. SiCtrl use control siRNA as negative control. *K–L*, HEK293T cells treated with DMSO or 10 μM Tub A (24 h) were infected with VSV-GFP, and viral titers were quantified (TCID_50_). β-actin, β-tubulin, and GAPDH were included as loading controls. GFP were normalized to loading controls. Statistical significance: ∗, *p* < 0.05, ∗∗, *p* < 0.01, ∗∗∗, *p* < 0.001.

**Fig. S3:**
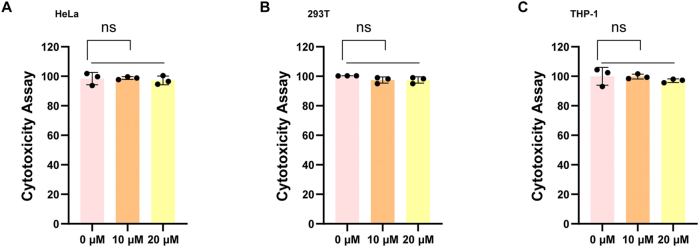


To further investigate HDAC6’s role, we overexpressed HDAC6 in HEK293T cells and observed enhanced VSV infection (Fig. 1, *A* and *B*). Conversely, Tub A treatment (10 or 20 μM) dose-dependently inhibited VSV replication (Fig. 1, *C* and *D*). Similar results were obtained in HeLa cells upon HDAC6 knockdown (si-HDAC6) or Tub A treatment (Fig. 1, *E*–*H*). These findings were corroborated in THP-1 cells (Fig. 1, *I* and *J*), with no cytotoxicity observed at tested concentrations (Fig. S3). A TCID_50_ assay confirmed that Tub A reduced VSV titers (Fig. 1, *K*–*L*), collectively demonstrating HDAC6’s involvement in VSV replication.

### HDAC6 physically interacts with STING

**Figure 2:**
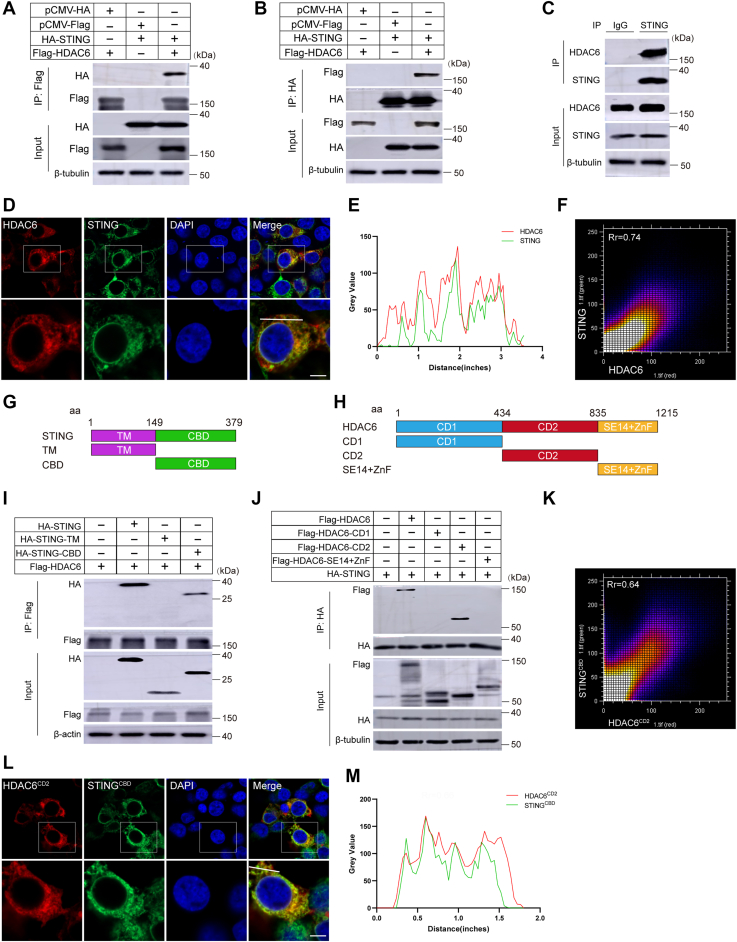
**HDAC6 physically interacts with STING**. *A–B*, HEK293T cells were co-transfected with empty vector or HDAC6-Flag and HA-STING. Cell lysates were subjected to co-immunoprecipitation (Co-IP) using anti-FLAG or anti-HA antibodies, followed by Western blotting with indicated antibodies. *C*, Endogenous interaction of HDAC6 and STING in THP-1 cells. Cell lysates were immunoprecipitated with anti-STING antibody (IgG as control) and probed for HDAC6 and STING. *D–F*, confocal microscopy of HEK293T cells co-expressing HDAC6-Flag (red) and STING-HA (*green*). Scale bar, 2 μm. Co-localization analysis (ImageJ): Plot profile confirmed overlapping fluorescence signals (E) Pearson’s coefficient (R) = 0.74 (F), indicating strong correlation (R scale: 0 = no correlation; 1 = perfect co-localization). *G*, domain mapping of human STING: N-terminal (Transmembrane Domain, 1–149 aa, *purple*) and C-terminal (cGAMP-Binding Domain, 150–379 aa, *green*). *H*, domain structure of human HDAC6: Deacetylase domains (Catalytic Domain 1, 1–434 aa, *blue*; Catalytic Domain 2, 435–835 aa, *red*) and C-terminal domain (SE14+ZnF, 836–1215 aa, *orange*). *I*, HDAC6 binds the C-terminal region of STING^CBD^: HEK293T cells were co-transfected with HDAC6-Flag + full-length STING-HA or HDAC6-Flag + STING truncations (TM or CBD). FLAG-IP followed by western blotting confirmed interaction with STING^CBD^. *J*, the HDAC6 CD2 domain mediates STING binding. HEK293T cells were co-transfected with STING-HA + HDAC6 truncations (CD1, CD2, and SE14+ZnF). FLAG-IP revealed HDAC6^CD2^ as the critical interaction domain. *K–M*, confocal microscopy of HEK293T cells co-expressing: HDAC6^CD2^-Flag (*red*) and STING^CBD^-HA (*green*). Scale bar, 2 μm. Pearson’s R = 0.64, confirming partial co-localization.

**Fig. S4:**
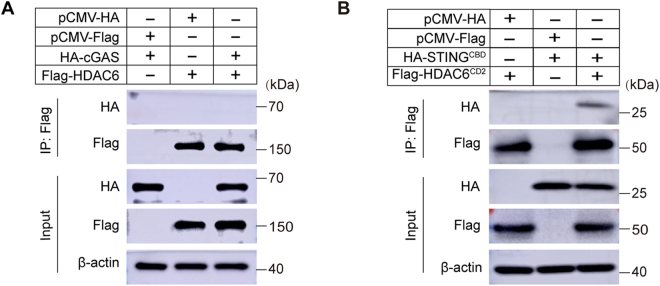


Given STING’s reported role in RNA virus-induced autophagy (38), we hypothesized that HDAC6 might regulate VSV replication *via* the cGAS-STING pathway. Co-immunoprecipitation (Co-IP) assay in HEK293T cells revealed a robust interaction between HDAC6 and STING (Fig. 2, *A* and *B*) but not cGAS (Fig. S4*A*). Endogenous interaction was confirmed in THP-1 cells (Fig. 2*C*), and immunofluorescence microscopy demonstrated colocalization (Fig. 2, *D*–*F*).

To map the interaction domains, we generated truncation mutants of STING (Fig. 2*G*) and HDAC6 (Fig. 2*H*). Co-IP assays identified the HDAC6 CD2 domain (Catalytic Domain 2, aa 435–835, encompassing its deacetylase domains) and the STING CBD (cGAMP-Binding Domain, aa 150–379) as critical for binding (Fig. 2, *I* and *J*, S4*B*). Microscopy confirmed colocalization of these domains (Fig. 2, *K*–*M*).

### HDAC6 deacetylates STING at K338

**Figure 3:**
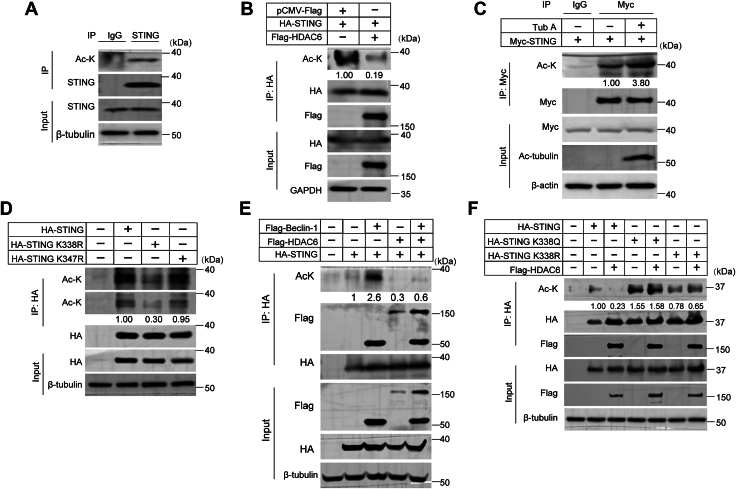
**HDAC6 deacetylates STING at K338**. *A*, endogenous STING and acetylation in THP-1 cells. Cell lysates were immunoprecipitated (IP) with anti-STING or control IgG antibodies, followed by immunoblotting for total STING and acetylated STING (ac-STING). 10% input shown as loading control. *B*, HDAC6-mediated STING deacetylation. HEK293T cells (2.5 × 10^6^) were co-transfected with HA-tagged STING and either empty vectors or Flag-tagged HDAC6 for 24 h. HA immunoprecipitates were analyzed for pan-acetylated lysines (pan-ac-K), Flag, HA, and GAPDH (loading control). *C*, Tubastatin A (Tub *A*) enhances STING acetylation. HEK293T cells (2.5 × 10^6^) expressing Myc-STING were treated with 10 μM Tub *A* or DMSO for 24 h. Myc immunoprecipitates were probed for pan-ac-K, Myc, and β-actin (loading control). IgG served as negative control. *D*, mapping STING acetylation sites. HEK293T cells were transfected with various HA-STING mutants. HA immunoprecipitates were analyzed for pan-ac-K and HA. *E*, HDAC6 rescues Beclin one from the acetylation of STING. HEK293T cells were co-transfected with Flag-HDAC6, Flag-Beclin-1, and HA-STING. HA immunoprecipitates were probed for pan-ac-K, Flag, and HA. *F*, functional characterization of STING acetylation mutants. HEK293T cells were co-transfected with Flag-HDAC6 and either HA-STING-WT, K338Q (acetylation mimic), or K338R (deacetylation-mimic). HA immunoprecipitates were analyzed for pan-ac-K, Flag, and HA. Pan-ac-K -STING were normalized to the total level of the protein.

**Fig. S5:**
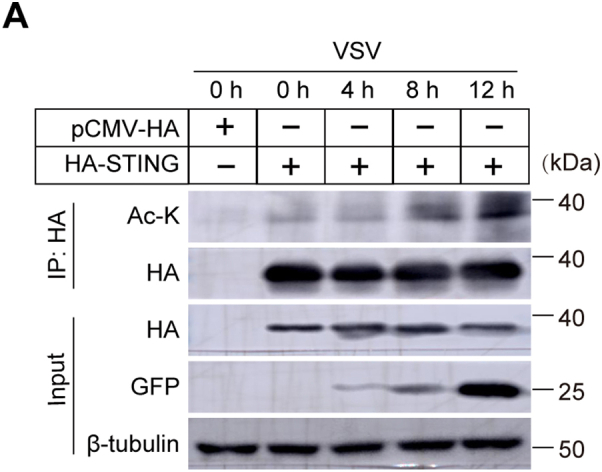


Since HDAC6’s deacetylase domain interacts with STING, we assessed whether HDAC6 regulates STING acetylation. Endogenous STING acetylation was detected in THP-1 cells (Fig. 3*A*). Recent studies have shown that acetylation of host or viral proteins plays an important role in virus adsorption, invasion, synthesis, assembly and release as well as host antiviral immune response (39). To investigate whether STING acetylation is associated with VSV infection, HEK293T cells were transfected with empty vector or STING-HA plasmids followed by infection with VSV for 0, 4, 8, or 12 h. STING was immunoprecipitated with HA antibody and the acetylation of STING was detected by immunoblotting with an anti-acetyl-lysine antibody. We found that STING acetylation gradually increased with the duration of VSV infection (Fig. S5), suggesting that increased STING acetylation may be a key step in the antiviral response. Next, cells were co-transfected with Flag-HDAC6 and HA-STING plasmids. Cell lysates were immunoprecipitated with HA antibody. The result showed that the acetylation of STING was significantly reduced following HDAC6 overexpression (Fig. 3*B*). To further confirm this data, the HDAC6-specific inhibitor Tub A was used to treat HEK293T, cells and the acetylation of STING was quantified. As shown in Figure 3*C*, STING acetylation increased when the cells were treated with Tub A compared with DMSO treatment. These data concluded that HDAC6 is a specific deacetylase of STING.

**Fig. S6:**
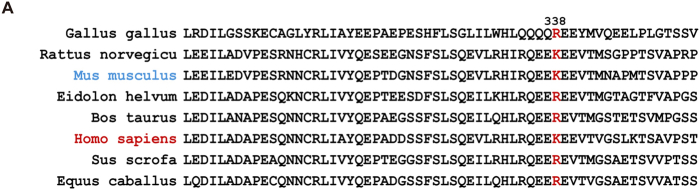


Mass spectrometry identified a single peptide (HLRQEEKEEVTVGSLK) containing K338 (Table 2), consistent with prior reports (12). We found that the STING K338R mutant exhibited significantly reduced acetylation (Fig. 3*D*). HDAC6 rescues Beclin one from the acetylation of STING, and HDAC6 could not deacetylate this mutant (Fig. 3, *E* and *F*), confirming K338 as the critical site. Notably, K338 is conserved in human and mouse STING but replaced by arginine in other species (Fig. S6).

**Table 2:** A list of the peptides of STING deacetylated by HDAC6

Sequence	IonScore	Charge	MH + [Da]	Abundance
ACLGCPLR	31	2	946.46	309280704
AGTCVLEYATPLQTLFAMSQYSQAGFSR	77	3	3097.48	82501424
AGTCVLEYATPLQTLFAMSQYSQAGFSR	86	3	3113.47	52402120
EDRLEQAK	33	2	988.51	885374016
EEVTVGSLK	59	2	961.52	538526336
GLAPAEISAVCEK	30	2	1344.68	483808288
GNFNVAHGLAWSYYIGYLR	50	3	2201.09	111094432
HLRQEEKEEVTVGSLK	44	4	1882	162207600
LIAYQEPADDSSFSLSQEVLR	131	2	2368.17	1334437160
LILPELQAR	45	2	1052.65	3752422564
LYILLPLDCGVPDNLSMADPNIR	68	2	2599.33	170672060
LYILLPLDCGVPDNLSMADPNIR	59	2	2615.32	14949504
QEEKEEVTVGSLK	37	3	1475.76	1822602112
TLEDILADAPESQNNCR	63	2	1945.89	271863776
TSAVPSTSTMSQEPELLISGMEK	20	2	2423.17	279761504
TSAVPSTSTMSQEPELLISGMEK	32	3	2439.16	10752824
TSAVPSTSTMSQEPELLISGMEK	52	2	2455.16	14070500
TSAVPSTSTMSQEPELLISGMEKPLPLR	31	3	2999.54	1329152128
TSAVPSTSTMSQEPELLISGMEKPLPLR	48	3	3015.54	830392192
TSAVPSTSTMSQEPELLISGMEKPLPLR	39	3	3031.53	438005024
TYNQHYNNLLR	35	3	1435.71	3016265984
VYSNSIYELLENGQR	97	2	1784.88	339526016

### STING deacetylation impairs TBK1 binding and STING phosphorylation

**Figure 4:**
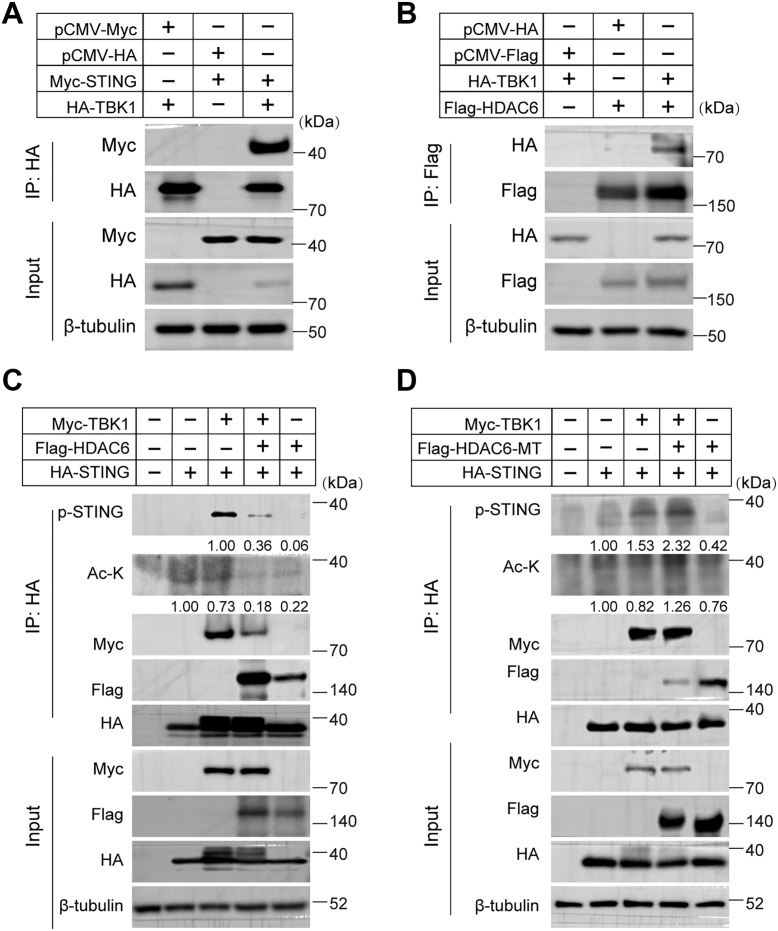
**STING deacetylation inhibits its phosphorylation**. *A*, TBK1-STING interaction. HEK293T cells were co-transfected with Myc-STING and HA-TBK1 for 24 h. Cell lysates were immunoprecipitated (IP) with anti-HA antibody and immunoblotted for the indicated proteins. *B*, HDAC6-TBK1 interaction. HEK293T cells expressing Flag-HDAC6 and HA-TBK1 were subjected to Flag-IP followed by immunoblotting to assess protein interactions. *C*, HDAC6-mediated suppression of STING phosphorylation. HEK293T cells were co-transfected with HA-STING, Flag-HDAC6, and Myc-TBK1. HA-IP samples were analyzed for STING phosphorylation (p-STING) and total proteins levels. *D*, catalytic activity requirement. HEK293T cells expressing HA-STING, catalytically inactive Flag-HDAC6-MT, Myc-TBK1 were analyzed by HA-IP to demonstrate the dependence on HDAC6 deacetylase activity. β-tubulin was included as loading control. Pan-ac-K -STING and p-STING were normalized to the total level of the protein.

It has been reported that the deacetylation of TBK1 by HDAC3 enhances the kinase activity of TBK1 (40). However, whether STING deacetylation by HDAC6 affects STING functions, such as phosphorylation, remains to be explored. To test this, cells were transfected with HA-STING, together with Flag-HDAC6 plasmids, and cell extracts were precipitated with HA antibody. We found that overexpression of HDAC6 resulted in the deacetylation of STING and decreased phosphorylation of STING at S366, whereas Tub A treatment increased STING acetylation and phosphorylation at S366 (Fig. S7), a site critical for TBK1 binding and IRF3 activation. Furthermore, according to previous reports, the C-terminal region of STING interacts with TBK1 and is phosphorylated by TBK1 at S366 in human cells (41, 42). To confirm this, we first validated the interaction between TBK1 and STING using the Co-IP assay (Fig. 4*A*). Additionally, HDAC6 was found to interact with TBK1 (Fig. 4*B*). To determine whether HDAC6-mediated deacetylation of STING affects its phosphorylation and binding to TBK1, cells were transfected with the following: empty vector (control), HA-STING alone, HA-STING + Myc-TBK1, HA-STING + Myc-TBK1 + Flag-HDAC6, or HA-STING + Flag-HDAC6. Cell lysates were immunoprecipitated with an HA antibody. The results showed that overexpression of HDAC6 impaired the phosphorylation at S366, and more interestingly, overexpression of HDAC6 decreased the binding of STING to TBK1 (Fig. 4*C*). Conversely, overexpression of a catalytically inactive HDAC6 (Flag-tagged HDAC6-MT) did not affect the phosphorylation of STING or its binding to TBK1 (Fig. 4*D*), which clearly demonstrated the essential function of the deacetylase activity of HDAC6 in regulating STING-mediated signaling pathway. The HDAC6 inactive mutant has been described previously (43, 44).

### STING acetylation status dictates antiviral responses

Phosphorylation dynamics of STING at serine 366 (S366) are tightly controlled by TBK1 kinase activity, which facilitates subsequent IRF3 recruitment and phosphorylation cascade (45). Recent studies have revealed that DSTYK, a dual-specificity kinase targeting both serine/threonine and tyrosine residues, synergizes with TBK1 to enhance STING phosphorylation specifically at late endosomal compartments (46). Our findings demonstrate that HDAC6-mediated deacetylation of STING significantly attenuates its phosphorylation status.

**Figure 5:**
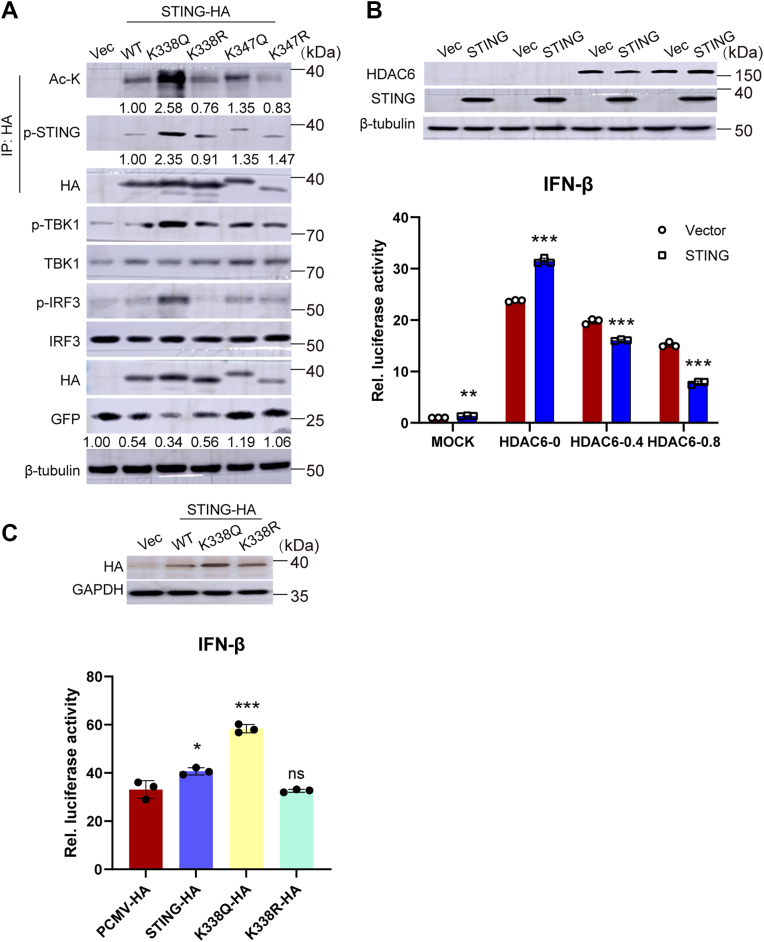
**STING acetylation at K338 enhances IFN-β production and restricts VSV replication**. *A*, STING acetylation-dependent protein interactions. HEK293T cells expressing STING WT or mutants (K338Q, K338R, K347Q, and K347R) were infected with VSV-GFP (12 h), followed by co-immunoprecipitation (Co-IP) to analyze acetylation-dependent protein complexes. Pan-ac-K -STING and p-STING were normalized to the total level of the protein. GFP were normalized to the loading control. *B*, HDAC6 dose-dependently suppresses IFN-β promoter activity. HEK293T-STING cells were co-transfected with IFN-β-luciferase reporter, pRL-TK (normalization control), empty vector or HA-STING, and increasing concentrations of Flag-HDAC6 (0, 0.4, and 0.8 μg) for 24 h. The cells were infected with VSV (12 h) or mock-treated, and luciferase activity was measured. Data represent the mean ± SD (n = 3). *C*, STING K338Q enhances antiviral signaling. HEK293T-STING cells were co-transfected with IFN-β-luciferase reporter, pRL-TK, and HA-tagged STING variants (WT, K338Q, and K338R) for 24 h. Following VSV infection (12 h), luciferase activity demonstrated that the acetylation-mimic K338Q significantly potentiated IFN-β induction compared to WT and deacetylation-mimic K338R. Data represent the mean ± SD (n = 3). Statistical significance: ∗, *p* < 0.05, ∗∗, *p* < 0.01, ∗∗∗, *p* < 0.001 Student’s *t* test.

To investigate this regulatory mechanism, we established a viral infection model transfected with STING mutants (K338Q, K338R, K347Q, K347R) followed by VSV challenge for 12 h for Co-IP assay. Notably, the STING K338Q (acetylation mimic) enhanced TBK1 phosphorylation kinetics and potent suppression of viral replication, whereas the STING K338R (deacetylation mimic) impaired IRF3 phosphorylation capacity with corresponding recovery of VSV replication (Fig. 5*A*). The results confirmed that STING acetylation status directly modulates its phosphorylation efficiency, revealing that a mimic of hyperacetylated STING (K338Q) amplifies S366 phosphorylation-dependent TBK1 activation to restrict viral propagation (Fig. 5*A*).

Further mechanistic exploration of STING post-translational modification cross-talk in innate immunity demonstrated that HDAC6-mediated deacetylation negatively regulates STING-induced IFN-β production. As shown in Figure 5*B*, under basal conditions, wild-type STING showed modest IFN-β induction capacity, but progressive HDAC6 overexpression following viral infection led to dose-dependent suppression of IFN signaling. Importantly, the above results reveal reciprocal regulatory patterns: HDAC6 promotes VSV replication through STING deacetylation, while STING K338Q sustains robust IFN-β production, STING K338R exhibits modestly impaired IFN-β production (Fig. 5*C*), and STING K338Q likely stabilizes its active conformation, enhancing TBK1 recruitment and phosphorylation efficiency. These findings establish a critical regulatory axis between STING acetylation and phosphorylation in modulating TBK1-IRF3 axis activation during viral infection.

## Discussion

While the cGAS-STING axis is well-established in DNA pathogen recognition, its involvement in RNA virus infections remains less defined. RNA viruses are typically detected by cytoplasmic RLRs (RIG-I and MDA5) *via* the mitochondrial antiviral signaling protein (MAVS) adaptor. For instance, HDAC6 enhances RIG-I sensing by deacetylating at Lys-909 (47). However, recent studies implicate the cGAS-STING pathway in modulating innate immunity against RNA viruses. STING knockout suppresses FMDV replication (38), and a 2024 review highlighted its context-dependent roles in SARS-CoV-2, HIV, and flaviviral infections (11). Intriguingly, HDAC6 exhibits dual immunoregulatory functions: it potentiates RIG-I activation while suppressing STING signaling during VSV infection.

As a class IIb deacetylase, HDAC6 governs diverse processes, including immunity, cytoskeletal dynamics, and protein degradation (48). It modulates viral lifecycles by deacetylating substrates like α-tubulin, HSP90, β-catenin, and TRIM21 (48). For example, HDAC6 restricts IAV trafficking by deacetylating microtubules (16) and inhibits IAV RNA synthesis *via* RNA polymerase PA subunit deacetylation (49). Paradoxically, HDAC6 also promotes IAV uncoating through dynein-mediated aggresome formation (24), underscoring its pleiotropic effects. Synthetic inhibitors (*e*.*g*., DARPins) targeting HDAC6’s ZnF domain disrupt aggresome formation and stress granule assembly, impairing viral replication (50).

We observed that HDAC6 knockdown or pharmacological inhibition (Tub A treatment) reduced VSV replication (Fig. 1). Given HDAC6’s reported role in degrading K48-ubiquitinated cGAS during porcine circovirus type 2 (PCV2) infection (51) and emerging evidence of cGAS-STING activity in RNA virus infections, we investigated HDAC6-STING interplay. Co-IP confirmed their physical interaction (Fig. 2). Our results are consistent with previous research, rabies virus M protein interacts with HDAC6 to activate the MEK/ERK signaling pathway and enhance RABV replication (52). We found that HDAC6 promotes VSV replication by interacting with STING and regulating downstream signaling pathways. Furthermore, the effect of MGCD-0103 on viral replication should also be taken into consideration. Our findings indicate that MGCD-0103 has an impact on the replication of HSV and VSV viruses (Fig. S2), suggesting that HDAC6 and class I HDACs significantly affect the replication of HSV/VSV. This is consistent with the known role of HDACs in viral infections. HDAC6 may play a role at different stages of the viral life cycle through its effects on cytoskeletal dynamics and innate immunity, while class I HDACs may do so by regulating viral gene expression. These findings do not contradict previous studies but highlight the complexity of the functions of HDAC subtypes during the infection process. Although the broad class I/HDAC11 inhibition of MGCD-0103 makes the mechanism attribution more complex, its extensive antiviral effect emphasizes the need for selective inhibitors targeting specific subtypes to dissect their individual roles. Further studies should validate these observations using genetic knockdowns or isoform-specific inhibitors to clarify the precise roles of HDAC6 and class I HDACs.

STING activity is regulated by PTMs, including phosphorylation (*e*.*g*., S366 for IRF3 recruitment) (45) and acetylation. Beclin-1 was recently shown to acetylate STING at K338, triggering its degradation (12). We found that VSV infection increased STING acetylation (Fig. S5), and mass spectrometry identified the acetylated peptide 'HLRQEEKEEVTVGSLK', suggesting HDAC6-mediated deacetylation at K338 (Fig. 3). Functional assays revealed that deacetylation of this residue is critical for STING activity.

According to the reports, dephosphorylation of ME1 at S336 by PGAM5 affects the acetylation of ACAT2 at K337 (53), and deacetylation of TRIM21 by HDAC6 affects the dimerization and ubiquitination of TRIM21, thereby influencing AdV virus replication (15). Furthermore, TBK1 interacts with STING and phosphorylates it at S366 to regulate downstream signaling pathways (42, 45, 54). Similarly, we discovered that HDAC6 competes with TBK1 for STING binding. This deacetylation reduces STING phosphorylation at S366 (Fig. 4), a key step in downstream signaling. Mutagenesis studies demonstrated that STING K338Q (acetylation-mimic) suppressed VSV replication, whereas K338R (a deacetylation-mimic) restored viral fitness (Fig. 5). Consistent with Figure 1, Tub A treatment increased STING acetylation and S366 phosphorylation, correlating with antiviral effects. These findings align with reports that HDAC6 depletion enhances cGAS-STING signaling and IFN production to restrict HSV-1 replication (26), suggesting a conserved mechanism wherein HDAC6 represses STING-driven immunity to benefit viral replication.

**Figure 6:**
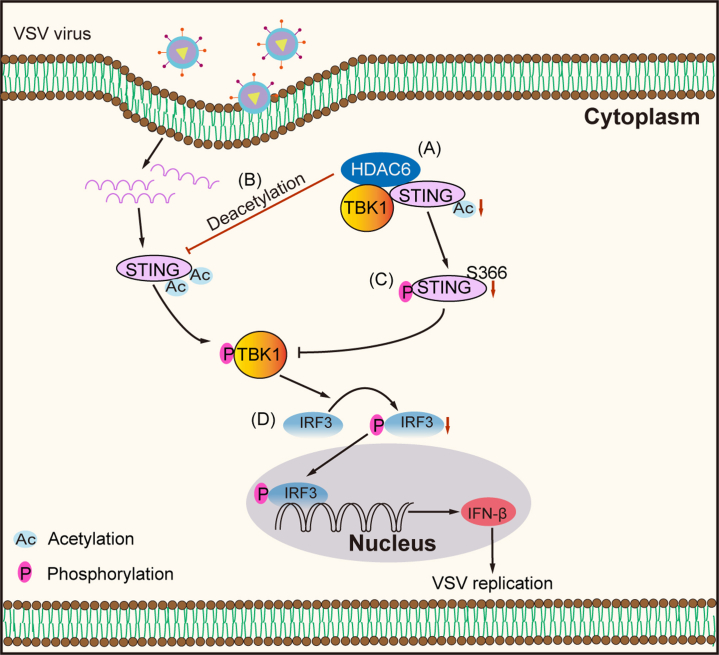
**Proposed mechanism of HDAC6-mediated regulation of cGAS-STING signaling and VSV replication**. Our findings demonstrate that HDAC6 functions as a critical regulator of antiviral immunity through the following mechanism: (*A*) Molecular interaction: HDAC6 directly binds to STING through its catalytic domain 2 (CD2, aa 435–835); (*B*) Deacetylation activity: HDAC6 deacetylates STING at Lys-338, removing critical acetyl groups; (*C*) Signaling modulation: Deacetylation of STING by HDAC6 to inhibit TBK1-mediated STING phosphorylation (p-STING) and attenuate downstream IFN-β production; and (*D*) Viral replication: This HDAC6-mediated suppression of STING activation creates a permissive environment that enhances VSV replication.

Our study elucidates a novel mechanism whereby HDAC6 deacetylates STING at K338, dampening phosphorylation-dependent activation to promote VSV replication (Fig. 6). Pharmacological blockade of HDAC6's deacetylase activity reversed this effect, highlighting its catalytic function as a linchpin in viral evasion.

HDAC6 inhibitors hold promise beyond virology, with roles in inflammation, oncology, and ciliopathies. For example, Tubacin, an HDAC6-selective inhibitor, inhibits viral replication by reducing viral RNA synthesis (55). Tub A, a selective HDAC6 antagonist, exhibits low neurotoxicity and multimodal efficiency: it suppresses pro-inflammatory cytokines (TNF-α and IL-6) (56), restores tumor-suppressive primary cilia (57), and inhibits viral RNA synthesis (31). Notably, HDAC6-knockout mice develop normally (58), supporting its safety as a therapeutic target.

In conclusion, we identify HDAC6 as a negative regulator of STING-mediated antiviral immunity and demonstrate the therapeutic potential of its inhibition. This work advances our understanding of PTM interplay in innate immunity and positions HDAC6 as a tractable target for infectious and inflammatory diseases.

## Experimental procedures

### Cells and viruses

HEK293T and HeLa cells were cultured in Dulbecco’s Modified Eagle Medium (DMEM). HEK293T-STING cells were maintained in DMEM supplemented with 1 μg/ml puromycin. THP-1 cells were grown in RPMI-1640 medium and differentiated into macrophages using 100 ng/ml phorbol 12-myristate 13-acetate (PMA) for 24 h. All media were supplemented with 10% fetal bovine serum (FBS) and 0.1% penicillin-streptomycin (v/v). Cells were incubated a 37 °C incubator with 5% CO_2_. The VSV-GFP/HSV-GFP strain was provided by our laboratory.

### Cytotoxicity assay

Cell viability was assessed using the CCK-8 assay (Solarbio, CA12010). Briefly, HEK293T cells were seeded in 96-well plates and incubated for 24 h before the addition of different concentrations of test reagents to each well. After 24 h, 10 μl CCK-8 reagent was added to each well and incubated for 2 h at 37 °C. Finally, absorbance was measured at 450 nm using a microplate reader.

### Plasmid transfection

HEK293T and THP-1 cells were transfected at 70 to 90% confluency using Lipo8000 (Beyotime) following the manufacturer’s protocol. Plasmid DNA-lipid complexes were prepared and added to cells.

### Lentivirus-mediated gene transfer

HEK293T cells were co-transfected with pLvx-mcherry-STING or scrambled control plasmids along with packaging vectors (psPAX2 and PMD2.G) using Lipo8000. Lentiviral supernatants were collected at 48 or 72 h post-transfection, filtered (0.45 μm), and used to infect HEK293T cells for 24 h. Stable cell lines were selected with 1 μg/ml puromycin for 7 days.

### Co-immunoprecipitation assays

The pCMV-Flag-HDAC6 and pCMV-Myc-STING plasmids are human constructs that were preserved in our laboratory. The truncation constructs of HDAC6 (HDAC6^1-434^ as CD1, HDAC6^435-835^ as CD2, HDAC6^836-1215^ as SE14+ZnF) and STING (STING^1-149^ as TM, STING^150-379^ as CBD) were subcloned into the pCMV-Flag or pCMV-HA expression vectors as we used previously (52). The primers used for PCR amplification are listed in Table S1. The HDAC6-MT plasmids (H216A and H611A) have been previously reported (43, 44, 47). HEK293T cells were transfected with the plasmids. After transfection, the cells were lysed in IP buffer (20 mM Tris, pH 7.5, 150 mM NaCl, 1% Triton X-100) containing protease/deacetylase/phosphatase inhibitors. The lysates were centrifuged (12,000 rpm for 15 min), and the supernatants were incubated with specific antibodies or IgG control (4 °C, overnight). Immune complexes were pulled down using Protein A/G magnetic beads, washed, and eluted with 1% SDS sample buffer for Western blotting.

### Indirect immunofluorescence assay

HEK293T cells were transfected with the indicated plasmids (24 h), fixed with 4% paraformaldehyde (30 min), and permeabilized with 0.5% Triton X-100. Cells were blocked with 3% BSA (1 h, room temperature), incubated with indicated primary antibodies (4 °C, overnight), and with fluorescent-conjugated secondary antibodies (37 °C, 30 min). The nuclei were stained with 4′, 6-diamino-2-phenylindole (10 min). Images were acquired using a Leica TSC SP8 confocal microscopy (Leica).

### HDAC6 knockdown

Chemically synthesized siRNA used in RNA interference (RNAi) assays was manufactured by Sangon. The knockdown of endogenous HDAC6 was carried out by transfection of the indicated HDAC6 siRNA into cells using jetPRIME polyplus reagents (Polyplus transfection, 101,000,046). The siRNA sequence targeted for si-HDAC6 is as follows: sense, 5′-CACCGUCAACGUGGCAUGGAA-3′, antisense, 5′-UUCCAUGCCACGUUGACGGUG-3′. The siRNA for control is as follows: sense, 5′-UUCUCCGAACGUGUCACGU/dT//dT/-3′, antisense, 5′-ACGUGACACGUUCGGAGAA/dT//dT/-3′.

### Western blotting

Target proteins were separated by SDS-PAGE and transferred to polyvinylidene fluoride membranes. The membranes were blocked with 5% skim milk (1 h, room temperature), incubated with primary antibodies (4 °C, overnight), and then incubated with HRP-conjugated secondary antibodies (1 h, room temperature). Signals were detected using ECL substrate (K-12045-D50) and imaged (GE-AI600, USA).

### TCID_50_ assay

Viral titers were determined by infecting HEK293T cells (96-well plates) with serially diluted supernatants. TCID_50_ was calculated after 24 h.

### Mass spectrometry

HEK293T cells were transfected with FLAG-HDAC6 and HA-STING plasmids for 24 h. The lysates were subjected to immunoprecipitation with HA magnetic beads at 4 °C overnight. HA beads-enriched complexes were subjected to SDS-PAGE gels, then stained using Coomassie brilliant blue (R-250) staining. After destaining, we put on new clean gloves and cut the target protein bands with a knife in the laminar flow cabinet and transfer them to a 1.5 ml centrifuge tube analyzed by mass spectrometry. Mass spectrometry analysis was provided by Jingjie PTM BioLab (Hangzhou) Co, Inc.

### RNA extraction and qRT-PCR

Total RNA was extracted from the cultured cells or tissues with TRIZOL reagent (Invitrogen) according to the manufacturer’s instructions, and the first-strand cDNA was reverse-transcribed with 5 × PrimeScript RT Master Mix (TakaRa). Quantitative PCR was performed using the TB Green Premix Ex Taq kit (Takara), and the primer sequences are listed in Appendix Table S1. The data were presented as the fold-change normalized to GAPDH based on the 2^−ΔΔCT^ method.

### Statistical analysis

Unpaired Student’s *t* test was used for statistical analysis of most experimental results in this study. Data are presented as the mean ± SD (n = 3). Student’s *t* test (GraphPad Prism 9.0) was used for comparison (*p* < 0.05 considered significant).

## Data availability

The authors confirm that all data underlying the findings are fully available without restriction. All relevant data are within the paper.

## Supporting information

This article contains supporting information (27, 59, 60, 61, 62, 63, 59, 60, 61, 62, 63).

## Conflict of Interest

The authors declare that they do not have any conflicts of interest with the content of this article.

## Figures and Tables

**Fig. S7:**