# Supplementary material for: Histone deacetylase 6 inhibits STING-dependent antiviral immunity via site-specific deacetylation
Source: J Biol Chem. 2025 Oct 23;301(12):110841. doi: 10.1016/j.jbc.2025.110841 (PMC12666567; doi:10.1016/j.jbc.2025.110841)
Supplement: Supporting Data 1 [file mmc1.docx]

SUPPORTING INFORMATION

**Histone Deacetylase 6 Inhibits STING-Dependent Antiviral Immunity via Site-Specific Deacetylation**

**Fig. S1.** Tub A treatment significantly inhibits VSV infection. (A-D) HeLa cells were infected with VSV/HSV at an MOI of 0.1/10. Cells were incubated for 1 h and treated with different HDACs inhibitors for the indicated concentrations. Samples were collected at 12 h post-infection. (E-H) HEK293T cells were treated with different HDACs inhibitors for the indicated hours and infected with VSV/HSV at an MOI of 0.1/10 for 12 h. (I-J) CCK-8 assay was used to detect the toxicity of different HDACs inhibitors on HEK293T cells. GFP fluorescence and WB blots represent the replication of VSV and HSV virus.

**Fig. S2.** MGCD-0103 inhibits replication of VSV and HSV. (A-B) HEK293T cells were treated with DMSO, 2, 4, 6, 8, 10 μΜ MGCD-0103 for 12 h, then infected with VSV/HSV at an MOI of 0.1/10 for 12 h. (C) CCK-8 assay was used to detect the toxicity of MGCD-0103 on HEK293T cells. (D-E) Effect of MGCD-0103 on VSV/HSV replication. The cells were treated with DMSO, 2, 4, 6, 8, 10 μΜ MGCD-0103 for 12 h, then infected with VSV/HSV at an MOI of 0.1/10 for 12 h. The VSV/HSV replication was detected by qRT-PCR.

**Fig. S3.** The cytotoxic assay of Tub A treatment in different cell lines.

**Fig. S4.** HDAC6^CD2^ interacts with STING^CBD^. (A) HEK293T cells were transfected with empty vector or HDAC6-Flag plasmid, and HA-tagged cGAS, and cell lysates were immunoprecipitated with FLAG antibodies, followed by a western blot with indicated antibodies. (B) HEK293T cells were transfected with empty vector or HDAC6^CD2^-Flag plasmid, and HA-tagged STING^CBD^, and cell lysates were immunoprecipitated with FLAG antibodies, followed by a western blot with indicated antibodies.

**Fig. S5.** Viral infection induced the acetylation of STlNG. HEK293T cells transfected with HA-EV or HA-STING for 24 h, then infected with VSV virus for 0, 4, 8, and 12 h, and the cells were harvested for Co-IP assay.

**Fig. S6.** Alignment of K338 locus in different species.

**Fig. S7.** Acetylation of STING affects the phosphorylation of STING at S366. (A) HEK293T cells were co-transfected with empty vector and HA-tagged STING, Flag-tagged HDAC6 and HA-tagged STING for 24 h, anti-HA immunoprecipitates were probed with indicated antibodies. (B) HEK293T cells were transfected with HA-tagged STING treated with DMSO or Tub A (10 μM) for 24 h, anti-HA immunoprecipitates were probed with the indicated antibodies.

Table S1. Primer sequences.

| **Primer name** | **Sequences** |
| --- | --- |
| **HDAC6-1F** | CGGGCTGCAGGAATTCATGGCTTTCTGTGCTAAAATGAG |
| **HDAC6-1R** | GCTTGATATCGAATTCTTACTCCTTTATTACAATGTGAG |
| **HDAC6-2F** | CGGGCTGCAGGAATTCATGTCTGTGGTCAGCATTAACAAG |
| **HDAC6-2R** | GCTTGATATCGAATTCTTACACCGTAATGGGGATGCCG |
| **HDAC6-3F** | CGGGCTGCAGGAATTCATGCTGTCTAGGCAAGAATGCATTG |
| **HDAC6-3R** | GCTTGATATCGAATTCTTATTTTAAAAGTGCTTCAAATC |
| **STING-1F** | CGGGATCCATGCCCCACTCCAGC |
| **STING-1R** | GCGTCGACACACACTGCAGAGATCTC |
| **STING-2F** | CGGGATCCGAAAAAGGGAATTTCAACGTGG |
| **STING-2R** | CCCAAGCTTTCAAGAGAAATCCGTGCG |
| **HSV-1-F** | GGAGATCCTTGCCCAGATGT |
| **HSV-1-R** | CTCTGCGTATTCCTCCGGAT |
| **VSV-F** | TGATAGTACCGGAGGATTGACGAC |
| **VSV-R** | CCTTGCAGTGACATGACTGCTCTT |
| **GAPDH-F** | ACCCACTCCTCCACCTTTG |
| **GAPDH-R** | CTCTTGTGCTCTTGCTGGG |

Table S2. The IC_50_ (μM) value of HDACs inhibitors.

|  | **Trichostatin A** | **Vorinostat** | **MGCD-0103** | **TMP269** | **Tubastatin A** | **PCI-34051** | **Nicotinamide** |
| --- | --- | --- | --- | --- | --- | --- | --- |
| **HDACs** | 0.0024 |  |  |  |  |  |  |
| **HDAC1** | 4.74 | 0.028 | 0.15 |  | 16.4 | 4 |  |
| **HDAC2** |  | 0.06 | 0.29 |  |  |  |  |
| **HDAC3** |  | 0.044 | 1.66 |  |  |  |  |
| **HDAC4** |  |  |  | 0.161 |  |  |  |
| **HDAC5** |  |  |  | 0.097 |  |  |  |
| **HDAC6** | 1.21 | 0.022 |  |  | 0.015 | 2.9 |  |
| **HDAC7** |  |  |  | 0.092 |  |  |  |
| **HDAC8** |  | 0.41 |  |  | 0.854 | 0.01 |  |
| **HDAC9** |  |  |  | 0.078 |  |  |  |
| **HDAC10** |  | 0.04 |  |  |  | 13 |  |
| **HDAC11** |  |  | 0.59 |  |  |  |  |
| **Sirtuins** |  |  |  |  |  |  | 50 |
| **References** | (59, 27) | (60) | (61) | (62) | (27) | (60) | (63) |
