# Supplementary material for: Histone deacetylase 6 inhibits STING-dependent antiviral immunity via site-specific deacetylation
Source: J Biol Chem. 2025 Oct 23;301(12):110841. doi: 10.1016/j.jbc.2025.110841 (PMC12666567; doi:10.1016/j.jbc.2025.110841)
Supplement: Supporting Data 2 [file mmc2.docx]

| **REAGENT or RESOURCE** | **SOURCE** | **IDENTIFIER** |
| --- | --- | --- |
| **Antibodies** |  |  |
| Mouse monoclonal anti-GFP | Abmart | M20004S |
| Mouse monoclonal anti-β-tubulin | Proteintech | 66240-1-Ig |
| Mouse monoclonal anti-actin | Proteintech | 66009-1-Ig |
| Rabbit polyclonal anti-GAPDH | Proteintech | 10494-1-AP |
| Mouse monoclonal anti-Flag | Abmart | M20008M |
| Mouse monoclonal anti-HA | Abmart | M20003M |
| Mouse monoclonal anti-Myc | Abmart | M20002M |
| Rabbit polyclonal anti-phosphorylated-Ser366-STING | Abmart | TA7416 |
| Rabbit polyclonal anti-acetyl-Histone H4-K12 | Abmart | T914227S |
| Rabbit monoclonal anti-HDAC6 | Cell signaling Technology | 7612S |
| Rabbit monoclonal anti-STING | Cell signaling Technology | 13647S |
| Rabbit monoclonal anti-phosphorylated-Ser172-TBK1 | Cell signaling Technology | 5483S |
| Rabbit monoclonal anti-TBK1 | Cell signaling Technology | 3504S |
| Rabbit monoclonal anti-phosphorylated-Ser396-IRF3 |  | 4947S |
| Rabbit monoclonal anti-IRF3 | Cell signaling Technology | 11904S |
| Rabbit polyclonal anti-acetylated-Lysine | Cell signaling Technology | 9441S |
| Goat anti-Mouse IgG (H+L) Secondary Antibody, HRP | ThermoFisher Scientific | 31460 |
| Goat anti-Rabbit IgG (H+L) Secondary Antibody, HRP | ThermoFisher Scientific | 31430 |
| Goat anti-Rabbit IgG (H+L) Highly Secondary Antibody, Alexa Fluor^TM^ 488 | ThermoFisher Scientific | A-11034 |
| Goat anti-Rabbit IgG (H+L) Highly Secondary Antibody, Alexa Fluor^TM^ 594 | ThermoFisher Scientific | A-11005 |
| **Chemicals, Enzymes and other reagents** |  |  |
| DMSO | Solarbio | D8371 |
| Tubastatin A (Tub A) | Selleck | S8049 |
| Vorinostat (SAHA) | Apexbio | C4018-50 |
| Trichostatin A (TSA) | Apexbio | A8183-1 |
| TMP-269 | MCE | HY-18360 |
| PCI-34051 | MCE | HY-15224 |
| MGCD-0103 | MCE | HY-12164 |
| Nicotinamide (NIC) | Selleck | S1899 |
| Phorbol 12-myristate 13-acetate (PMA) | MCE | HY-18739 |
| Puromycin | Solarbio | P8230-25 |
| Anti-HA Magnetic Beads | HUABIO | HAK21042 |
| [Anti-DYKDDDDK (Flag) Magnetic Beads](javascript:;) | HUABIO | HAK21011 |
| Protein A/G-Agarose | Abmart | A10001M |
| Endofree Maxi Plasmid Kit | Omega | D6915-03 |
| **Oligonucleotides and other sequences-based reagents** |  |  |
| PCR primers | This study | Table S1 |
| **Recombinant DNA** |  |  |
| pCMV-3 x Myc-TBK1(human)-Neo | MIAOLING BIOLOGY | P63897 |
| pCMV-HDAC6-MT (human)-3 x FLAG-Neo | MIAOLING BIOLOGY | P56050 |
| pCMV-Beclin-1 (human)-3 x FLAG-Neo | MIAOLING BIOLOGY | P60982 |
| **REAGENT or RESOURCE** | **SOURCE** | **IDENTIFIER** |
| **Tools** |  |  |
| GraphPad Prism 9.0.0 | https://www.graphpad.com/ |  |
| ImageJ _v1.8.0 | <https://www.imagej.net/ij/> |  |
